# *In Vitro* Antimalarial Susceptibility of *Plasmodium falciparum* and *Plasmodium berghei* Isolates to Selected Antimalarial Agents, Column Chromatographic Subfractions of *Glyphaea brevis* Leaves Extract and FTIR and GCMS of SF8

**DOI:** 10.21315/tlsr2023.34.2.14

**Published:** 2023-07-21

**Authors:** Tayo Micheal Anjuwon, Joseph Olorunmola Ehinmidu, Kola Matthew Anigo, Dorcas Bolanle James

**Affiliations:** 1 Department of Biochemistry, Ahmadu Bello University, P.M.B 1069 Zaria, Kaduna State, Nigeria; 2 Department of Pharmaceutical Microbiology, Ahmadu Bello University, P.M.B 1069 Zaria, Kaduna State, Nigeria; 3 Department of Biochemistry, University of Abuja, FCT, Nigeria

**Keywords:** Malaria, *Plasmodium falciparum*, *Plasmodium berghei*, FTIR, GCMS, Susceptibility

## Abstract

Malaria still remains a life-threatening parasitic disease with universal targets set for control and elimination. This study aimed to evaluate the *in vitro* antimalarial susceptibility of *Plasmodium falciparum* isolates and *Plasmodium berghei* to selected antimalarial agents and column chromatographic subfractions of *Glyphaea brevis* leaves extract and FTIR and GCMS of SF8. Trager and Jensen as well as World Health Organisation (WHO) standardised *in vitro* micro-test system methods were used to determine susceptibility on the patients’ blood samples; Column chromatographic procedure was carried out to obtain 11 pooled fractions; FTIR and GCMS were used to determine functional groups and phytochemicals respectively. *In vitro* anti-plasmodial activity against *P. falciparum* clinical isolates had IC_50_ range of 1.03 μg/mL–7.63 μg/mL while their IC_50_ against *P. berghei* ranges from 4.32 μg/mL–7.89 μg/mL. Subfraction 8 (SF8) had the least IC_50_ of 4.32 μg/mL. The FTIR spectrum showed the presence of isoprenoid, alcohol, phenol, alkane, alkenes, ester, carboxylic acids, aromatics and nitro compounds while GCMS identified dodecanoic acid, methyl ester; carotol; hexadecanoic acid, methyl ester; 9-octadecenoic acid (Z)-, methyl ester (oleic acid); methyl stearate; heptadecanoic acid, 16-methyl-, methyl ester; all with their antimalarial reported activities. In conclusion, *G. brevis* has a great potential for drug development against malaria parasite since it inhibited schizont growth and possesses phytocompounds with antimalarial report.

**Highlight**

- *In vitro* anti-plasmodial activity against *P. falciparum* clinical isolates had IC_50_ range of 1.03 μg/mL–7.63 μg/mL while their IC_50_ against *P. berghei* ranges from 4.32 μg/mL–7.89 μg/mL. Subfraction 8 (SF8) had the least IC_50_ of 4.32 μg/mL.
- The FTIR spectrum showed the presence of isoprenoid, alcohol, phenol, alkane, alkenes, ester, carboxylic acids, aromatics and nitro compounds.
- GCMS identified Dodecanoic acid, methyl ester; Carotol; Hexadecanoic acid, methyl ester; 9-Octadecenoic acid (Z)-, methyl ester (Oleic acid); Methyl stearate; Heptadecanoic acid, 16-methyl-, methyl ester; all with their antimalarial reported activities.
- *G. brevis* has a great potential for drug development against malaria parasite since it inhibited schizont growth and possesses phytocompounds with antimalarial report.

## INTRODUCTION

Malaria still remains one of the major and most devastating human parasitic infectious diseases in the world, responsible for high rate of morbidity and mortality in developing countries (Tindana *et al*. 2021). An estimate of 241 million cases of malaria and 627,000 malaria deaths occurred globally in 2020 (WHO 2022). Millions of deaths attributable to malaria are still being recorded with a huge epidemiologic burden in Africa and continues to cripple the economic development in the region. In Nigeria, malaria is responsible for 60% outpatient visits to health facilities, 30% childhood death, 25% of death in children under one year and 11% maternal death. Also, the financial loss due to malaria annually is estimated to be about by 132 billion Naira (USD318 million) in form of treatment costs, prevention, loss of man-hours and so on (Federal Ministry of Health Nigeria 2017); globally, it has been estimated that USD5.6 billion is required annually to remain on track toward WHO global malaria strategy targets (Oladipo *et al*. 2022)

The constant evolution of the malaria parasite has rendered the cheapest and most widely available antimalarial treatments ineffective, especially with the reports on the increasing resistance of *Plasmodium falciparum* to currently used artemisinin-based compounds (Cui *et al*. 2012; Nassirou *et al*. 2015). There is great concern that malaria parasite may soon develop total resistance to modern approach, thereby making medicinal plants to generate recent attention in the treatment of malaria. There is an urgent need to explore the naturally endowed rich biodiversity of communities through research that could translate to benefits for mankind (Olasehinde *et al*. 2014).

*Glyphaea brevis* (*G. brevis*) belongs to the family Tiliaceae; it is a spreading shrub, climber or small tree up to 8 m high. It is very common in undergrowth of closed forest, secondary jungle and on riverbanks, lowlands to sub-mountain and widespread in tropical Africa and South America. It is considered a vegetable in some cultures (Anjuwon *et al*. 2015; Okafor *et al*. 2016). Traditionally *G. brevis* is used in Africa and South America to treat various disease conditions of man including fevers, gonorrhoea, dysentery, stomach troubles, lung troubles, parasitic infections, convulsions and constipation. N-butanol fraction of methanol crude extract (NBFx-MExt) of *G. brevis* leaves has showed suppressive antiplasmodial activity against *Plasmodium berghei* infected mice (Anjuwon *et al*. 2015).

In recent years, *G. brevis* has come under the limelight of researchers in various parts of the world due to its broad ethno-medicinal uses. Its crude extracts and phytochemicals showed a wide spectrum of activity including anti-infective, antioxidant, anti-allergic, anti-inflammatory, anticonvulsant, hypocholesterolaemic activity, weight control and blood glucose control activity, glycosidase inhibitory activity and hepatoprotective effect (Osafo & Boakye 2016).

Therapeutics based on combinations with artemisinin and derivatives (ACTs), perhaps the most effective treatment against *falciparum* malaria recommended by the World Health Organisation (WHO) emerged as the best practical solution in overcoming the resistance of select strains (WHO 2008). The multidrug-resistant strains of *Plasmodium* represent a great threat in relation to the treatment and prevention of the disease leading to limitations in drugs application and current primary cause of control failure (Akuodor *et al*. 2017). This infectious disease is highly fatal, and may predispose the patient to opportunistic infections unless diagnosed and treated in a timely fashion (Nardos & Makonnen 2017).

The use of medicinal plants in modern medicine is less valued due to the fact that though hundreds of plants are used in the world to prevent or to cure diseases, scientific evidence in terms of modern medicine is lacking in some cases. Since its N-butanol fraction has been shown to have suppressive antiplasmodial effect (Anjuwon *et al*. 2015), further purification method is necessary if the plant is to be considered for drug design and development.

## METHODOLOGY

### Plant Sample Collection, Extraction and Chromatographic Processes

Fresh leaves of *G. brevis* was collected from Irun Akoko, Ondo State and was brought for identification at the herbarium unit of Botany Department, Ahmadu Bello University, Zaria where a voucher number 2634 was obtained and specimen deposited; the air dried and powdered *G. brevis* leaves extraction procedures have been earlier reported (Anjuwon *et al*. 2019) to obtain N-butanol fraction of methanol crude extract (NBFx-MExt).

A preliminary thin layer chromatography (TLC) was carried out on the NBFx-MExt of *G. brevis* leaves to determine the best solvent system that would give the highest number of distinct band of components. A pre-coated aluminium chromatographic plate with silica gel was used. The NBFx-MExt was dissolved in the solvent used for extraction and was applied several times on the plate using a micro haematocrit capillary tube until the load quantity was adjusted sufficiently for the experiment. The plate was placed in a chromatographic tank and eluted with a varying ratio of the different solvent system as the mobile phase in order to get the suitable solvent. Thereafter the plate was removed, dried in air and developed using 20% sulphuric acid in methanol to identify most suitable solvent system.

Column chromatography of the NBFx-MExt, as a follow up from the TLC plate result, was carried out. The column was conditioned using best solvent system with distinct bands (from the TLC analysis previously carried out) to pack the silica gel of 50 μm–200 μm mesh size. The NBFx-MExt (2.0 g) of *G. brevis* leaves was emptied into a porcelain mortar, 5 g of silica gel was added and a pestle was used to macerate the mixture to homogeneity. The mixture was carefully packed on top of the column chromatography and was then eluted with the varying solvents system. Approximately 50 mL/18 min flow rate was maintained. The collected elutes (fractions) were evaporated to near dryness. Each of the fractions was weighed and spotted on TLC plate, it was viewed under UV radiation. The plate was further sprayed with 20% sulfuric in methanol solution and dried for 15 min at 110°C (Ejele *et al*. 2012). This TLC procedure enabled similar fractions to be pooled together based on the pattern and retention factor (R_f_) values of the spots on the TLC plate into main fractions respectively. The R_f_ values were calculated using this formula:


$${\text{R}}_{\text{f}}=\text{Dc}/\text{Ds}$$

where Dc is distance travelled by component and Ds is distance travelled by solvent front.

The combined yield of fractions with the same R_f_ value and combined yield as percentage (%) of the NBFx-MExt fractionated were calculated. The intermediate pooled fractions were kept in a refrigerator for further analysis.

### Malaria Patients Blood Sample Collection and Study Protocol

This study was carried out at Department of Pharmaceutical Microbiology, A.B.U Zaria, Nigeria. The clinical isolates of *P. falciparum* were obtained from patients attending A.B.U Medical Service Centre. Blood samples of volunteered patients presented to the parasitology laboratory for malaria test with symptoms of uncomplicated malaria, mono infection with *P. falciparum* and parasitaemia level (1,000–80,000) asexual parasites per micro litre of the blood were recruited for the *in vitro* study were collected via venipuncture by Hospital Clinicians/Laboratory Scientist and dispensed in sterile EDTA tubes. The collected clinical blood samples were transported immediately to Pharmaceutical Microbiology laboratory, A.B.U Zaria for further analysis. The *in vitro* susceptibility test was carried out according to the standard culture techniques of Trager and Jensen (1976) and Mark III micro-test (Singh *et al*. 2015; WHO Malaria Unit 2001) with some modifications under strict aseptic condition.

*Plasmodium berghei* [susceptible (NK–65) strain] was obtained from National Institute of Medical Research (NIMR), Lagos, Nigeria. *P. berghei* has been used for *in vitro* assay as reported by Longley *et al*. (2015). The *berghei* parasite was passage into an albino mouse and continuous check and parasite maintenance were ensured.

### Antimalarial Drugs

Chloroquine (Sigma Aldrich), Artemether (Tianjin Kingyork Group Hubei Tianyao Pharmaceutical); Artemether/Lumenfantrine (Coartem**®** 80/480 mg: Novartis Pharmaceuticals); Artemisinin/piperaquine (Artequick® 62.5/375 mg: Artepharm Co.).

### Stock Solutions of Selected Antimalaria Drugs, N-butanol Fraction and Sub-Fractions Preparation

The N-butanol fraction and sub-fractions samples were dissolved in sterile distilled deionised water to obtain the highest working concentration of 160 μg/mL according to reported IC_50_ 3.60 μg/mL (Lawal *et al*. 2015). Stock solutions of Chloroquine, Artemether, Artemether-Lumefantrine (Coartem®) and Artemisinin-piperaquine (Artequick®) (standard controls) were prepared in 70% ethanol and distilled deionised water, respectively. All stock solutions were sterilised by filtration through 0.22 μm microfilters.

### Susceptibility Assay

Antimalarial drug susceptibility assay was carried out using the method of (Singh *et al*. 2015) adopted from WHO Malaria Unit (2001) with slight modification. For *in vitro* assay, 10 μL of the antimalarial agents was dispensed into wells B-F of graded concentration (well A was without drug). The solutions were left to dry to powder. Ninety microliters (90 μL) of complete culture medium (RPMI 1640 and O^+^ serum) was added into each well (A–F), then finally 10 μL of the parasitised erythrocytes dispensed into each of the wells.

For the extract, graded solutions of the *G. brevis* subfractions was prepared in sterile distilled deionised water and 10 μL each was also loaded into the microtiter well plate. Well B–F had the graded concentration of the extracts in the increasing doubling concentration from B to F, where A is the control wells that contain the patients’ blood and complete culture medium in ratio 1: 9 (10 μL: 90 μL) after blood samples were standardised and the extract well had the mixture of graded concentrations of the extracts, parasitised blood samples and complete culture medium in ratio 1:1:8 (10 μL:10 μL:80 μL) from well B through F. All these were done under strict aseptic condition.

All the plates were shaken gently without lifting it from the laboratory bench to properly mix the contents of the wells. The plates were incubated (5% CO_2_) at 37°C for 30 h (Trager & Jensen 1976). After incubation, the red cells decant were transferred to a clean grease free microscope slide by removing the supernatant from each well. The smears were allowed to dry for 24 h and stained with 5% Giemsa for 30 min.

### Microscopic Examination

Thick and thin films were prepared for microscopic examination of the *Plasmodium* species. Thin films were fixed with methanol for 60 sec, rinsed gently and allowed to dry. Both blood films were stained with 10% Giemsa of pH 7.1 for 30 min (WHO Malaria Unit 2001). Films were allowed to air-dry and were observed microscopically using x100 (oil immersion) objective lens as described by Cheesbrough (2000). The thin films were used to identify the parasites species while the thick films were used to determine the parasite density. Average parasite counts per 10 microscopic fields were determined and multiplied by a factor of 500 to estimate the number of parasite per microliter of blood (Greenwood & Armstrong 1991). The parasitaemia level within 1,000–80,000/μL of samples were recruited for the *in vitro* antimalarial susceptibility test (WHO Malaria Unit 2001).

### Post Culture Slides Examination

The stained thick films were examined under oil immersion objective (×100) lens and parasite densities (number) were counted and related with the parasite density in the control well. Schizont growth inhibition per 200 asexual parasites was counted in 10 microscopic fields. The control parasites culture freed from *G. brevis* subfractions or drugs were considered as 100% growth. The percentage inhibition per concentration was calculated using the formula (Ngemenya *et al*. 2006; WHO Malaria Unit 2001):


% inhibition per concentration=(% parasitaemia in control wells-% parasitaemia of the control)×100

The inhibitions were fed into the IC_50_ values, the concentration required to inhibit schizont growth by 50% were determined by linear regression analysis using Microsoft Excel 2016 from the schizont growth inhibition curves (Log of concentration versus percent inhibition) generated from each parasite-plant fraction interaction (Mustofa *et al*. 2007). Drug resistant *P. falciparum* parasites were identified with IC_50_ values greater than the peak plasma concentration of the antimalarial drugs.

### Fourier Transform Infrared Spectrophotometer (FTIR) Analysis

The IR transmission spectra of the SF8 were obtained using an FTIR spectrophotometer (Shimadzu, Japan). The powdered sample was loaded into FTIR spectroscope and the characteristic peaks were recorded using the FTIR spectrophotometer (Dwivedi *et al*. 2021).

### Gas Chromatography Mass Spectroscopy (GC-MS) Analysis

The subfraction 8 of *G. brevis* leaves extract with the least IC_50_ was accurately weighed and dissolved in methanol as the solvent. GC-MS experiment was then carried out using standard procedure (Waheed 2012).

### Ethical Consideration

Ethical clearance was obtained from A.B.U Zaria Health Service Ethical committee. Written informed consent was obtained from patients prior to collection of their blood samples for this study.

## RESULTS

### Thin Layer Chromatograph

Screening for suitable solvent system and combinations for column chromatography was carried out; hexane (100%), hexane: ethyl acetate (8:2; 1:1), ethyl acetate: methanol (1:1); ethyl acetate and methanol gave the best resolution. These were used by further varying their combination ratios.

### Column Chromatograph of N-butanol Fraction of *Glyphaea brevis* Leaves Extract

Ten grams (10 g) of N-butanol fraction was loaded on 100 g of silica gel (size 60 μm–120 μm) mesh and packed in column of 50 cm length, diameter of 2 mm. The solvent system combination used were: n-hexane (100%); n-hexane:ethyl acetate (4:1, 3:2, 2:3, 1:4); ethyl acetate (100%); ethyl acetate:methanol (4:1, 3:2, 2:3, 1:4), and methanol (100%); approximately 20 mL/5 min flow rate was maintained. The number of various solvents ran in order: 1–15, 16–30, 31–45, 46–55, 56–65, 66–75, 76–85, 86–95, 96–105, 106–115, 116–125, 126–137 as presented in Table 1.

The total of 137 collected fractions were evaporated to near dryness. Each of the fractions were spotted on TLC plate using ethyl acetate: methanol (9:1) as the mobile phase which was used for the pooling of the fractions, it was developed and viewed under UV radiation (λ = 254 nm and 365 nm). The plate was further sprayed with 20% vanillin sulfuric acid and dried for 15 min at 110°C. This TLC procedure enabled similar fractions to be pooled together based on the colour, movement pattern and shapes on the TLC plate. A total of 11 subfractions was then obtained, the range of the retention factors (RF) was between 0.38 and 0.81; SF5 had the highest yield (0.98 g) from the chromatographic runs.

### *In Vitro* Susceptibility Pattern of Antimalarial Agents

The screening of the N-butanol fraction and the 10 subfractions obtained from the column chromatography of the fraction was done using *Plasmodium falciparum* and *P. berghei* to determine their *in vitro* susceptibility pattern.

The IC_50_, i.e., the concentration of the test antimalarial agent that inhibited 50% of the parasite were calculated and compared their peak plasma concentration to determine the susceptibility of each drug and subfractions. Eight (8) blood samples obtained from patients were first screened to obtain three (3) chloroquine (CQ)-resistant clinical isolates. The three (3) CQ-resistant clinical isolates had peak plasma concentrations 6.56, 5.11 and 6.95 μmol as against 4.47 μmol for CQ as presented in Table 2.

The subfractions of N-butanol fraction of *G. brevis* leaves showed good anti-plasmodial activity *in vitro* against *P. falciparum* clinical isolates with IC_50_ range of 1.03 μg/mL–7.63 μg/mL while their IC_50_ against *P. berghei* ranges from 4.32 μg/L–7.89 μg/L as presented in Table 3. Subfraction 8 (SF8) had the least IC_50_ of 4.32 μg/mL, hence, it was considered for the FTIR and GCMS studies.

### Fourier Transform Infrared (FTIR) Spectroscopy Peak Values and Functional Groups of SF8 of *G. brevis* Leaves Extract

The SF8 of *G. brevis* leaves extract was analysed for functional groups identification using the FTIR spectroscopy. The FTIR spectra of the SF8 of *G. brevis* leaves extract as presented in the Table 4 shows the absorption peak characteristic of SF8 at a particular wavenumber, the functional groups and the corresponding bonds with movement. The FTIR spectrum showed the presence of isoprenoid, alcohol, phenol, alkane, alkenes, ester, carboxylic acids, aromatics and nitro compounds.

### Gas Chromatography Mass Spectrometry Analysis of the Sub-Fraction 8 of N-Butanol Fraction of *G. brevis* Leaves Extract

The results of the GC-MS analysis identified compounds that are present in the SF8 of N-butanol fraction of *G. brevis* leaves extract. The various components, their peak area and retention time (RT) as well as their reported biological activities are presented in the Table 5. The compounds identified by GC-MS were carvone with retention time (RT) of 7.56 min and molecular weight of 150.32 g/mol; dodecanoic acid, methyl ester (molecular weight: 214.34 g/mol); carotol (molecular weight: 222.37 g/mol), hexadecanoic acid, methyl ester (molecular weight: 270.45 g/mol); 9-octadecenoic acid (Z)-, methylester (oleic acid) (molecular weight: 296.50 g/mol); methyl stearate (molecular weight: 298.50 g/mol); heptadecanoic acid, 16-methyl-, methyl ester (molecular weight: 298.50 g/mol); clindamycin (molecular weight: 424. 98 g/mol); ropivacaine (molecular weight: 274.4 g/mol); l-alanine, n-propargyloxycarbonyl-, octadecyl ester, 3,4-methylenedioxypyrovalerone (molecular weight: 275.0 g/mol) and α-pyrrolidinopentiophenone (molecular weight: 231.0 g/mol).

## DISCUSSION

Malaria still remains a life-threatening parasitic disease that has been the highpoint of many nations of the world’s declarations, with universal targets set for control and elimination since more than two decades (Teng *et al*. 2019).

Screening of plants has become an attractive way of recognising new lead candidates for drug development against parasitic diseases. Plants are used as bases to the development of new synthetic/semi-synthetic drugs (Banzragchgarav *et al*. 2021).

This research explored *in vitro* antimalarial activity of the various column chromatographic subfractions (SFs) obtained from N-butanol fraction of *G. brevis* leaves extract against *P. falciparum and P. berghei* infection. Of all the subfractions that were evaluated, SF8 had the minimum inhibitory concentration (IC_50_) of 4.32 μg/mL. The subfractions of N-butanol fraction of *G. brevis* leaves showed good *in vitro* anti-plasmodial activity against *P. falciparum* clinical isolates with IC_50_ range of 1.03 μg/mL–7.63 μg/mL which is classified under active (0.03 < IC_50_ < 2.5) to moderately active (2.5 < IC_50_ < 10); their IC_50_ against *P. berghei* ranges from 4.32 μg/mL–7.89 μg/mL as moderately active (2.5 < IC_50_ < 10) (Eseyin *et al*. 2021). These IC_50s_ obtained in this research were very similar but lower than the reported values in Laryea *et al*. (2021).

*G. brevis* leaves N-butanol fraction and subfractions showed inhibitory effects on chloroquine resistant isolates of the malarial parasite hence giving a clear indication that the plant extract showed anti-plasmodial effects. This concurred with a study done by Marie *et al*. (2018) that presented three plants extracts having noteworthy anti-plasmodial activities, with good selectivity against chloroquine-sensitive and -resistant strains of *P. falciparum*.

Phytochemical investigations on medicinal plants like *G. brevis* have shown that it contains phenolic compounds, such as flavonoids, flavones and lignans, have been frequently investigated for their antimalarial activities and found to exhibit both *in vitro* and *in vivo* effectiveness against *Plasmodium* strains (Mamede *et al*. 2020; Shen *et al*. 2020). Thus, the expected outcome obtained from the study, being therapeutic in their effects against malaria parasites; agreeing with the research of Banzragchgarav *et al*. (2021).

FTIR spectroscopy is a rapid, non-destructive and high-resolution analytical technique used for the identification of various functional groups or chemical bonds found in plant extracts (Kalaichelvi & Dhivya 2017). The wavelength of light absorbed was the relevant feature of chemical bond as can be seen in the annotated spectrum. By interpreting the infrared absorption spectrum, the chemical bonds in a compound can be determined. Moreover, FTIR spectroscopy is an established time-saving method to characterise and identified functional groups (Kalaichelvi & Dhivya 2017).

Thus, in this research, FTIR technique was used to assess the IR fingerprint in SF8 of *G. brevis* leaves extract.

FTIR analysis revealed the presence of hydroxyl, phenols, alkanes, esters, carboxylic acids and nitro compounds that are vital functional groups in majority of pharmaceutically active phyto-constituents as also reported in the study of Dwivedi *et al*. (2021). The analysis revealed the presence of polyphenols and flavonoids due to O-H stretching, terpenes due to C-H group (Maobe *et al*. 2013); the presence of alkanes confirm the presence of alkaloids (Pawar & Kamble 2017) in *G. brevis*. All these compounds belong to secondary plant metabolites. The existence of characteristic functional groups of carboxylic acids, alcohols, phenols, esters, etc. could be responsible for the various medicinal properties of *G. brevis* (Baltacıoğlu *et al*. 2021; Kalaichelvi & Dhivya 2017). Studies also reported that these groups are present in plants such as leaves extract of *V. negundo* L. (Dwivedi *et al*. 2021; Janakiraman & Jeyaprakash 2015; Pawar & Kamble 2017).

The chemical constituents of SF8 of N-butanol fraction of *G. brevis* leaves extract were analysed by GC-MS, revealed the presence of 12 components. The GC-MS running time was 38.44 min, and 12 components were detected. The GC-MS spectra showed high concentration of fatty acids. Unsaturated fatty acids are not produced in humans but have been shown to possess diverse pharmacological properties and aid in general functioning of the human body. In the study of Orabueze *et al*. (2020), they reported on antimalarial properties of polyunsaturated fatty acids, especially the essential fatty acids and their bio-activity increases with their degree of unsaturation. Polyunsaturated fatty acids such that were identified in SF8 (Hexadecanoic acid, methyl ester; 9-octadecenoic acid methyl ester and 9-Octadecenoic acid (Z)-, methyl ester (Oleic acid)) were among the identified compounds in their active antiplasmodial fractions of their plant extract (Orabueze *et al*. 2020). Specifically, 9-Octadecenoic acid (Z)-, methyl ester and other ester compounds have been reported to possess anti-gastric, anti-(breast) cancer, antioxidant, anti-inflammatory, antiandrogenic and anemiagenic properties (Duraisamy & Selvaraju 2020; Lalitha & Palani 2017). For Oleic acid, it has been reported to have an anti-inflammatory potential through the activation of different pathways of immune competent cells, they also lower heart attack risk, artherosclerosis, and improved the effectiveness of herceptin which aids in cancer prevention; it also produces a suppression of lymphocyte proliferation, an inhibition of cytokine production and a reduction in Jurkat cells activity (Oche *et al*. 2017). It has been suggested that oleic acid causes disruptions of the plasma membrane, as well as depolarization and consequent breaching and activates an influx of calcium into the cells (Zahara *et al*. 2022).

Carvone, has been chosen as starting material for the synthesis of antimalarial agent Artemisinine, a new lactone sesquiterpene containing one peroxide binding (Araujo *et al*. 1991). *G. brevis* may possibly be a good source for its extraction. Also, carotol is sesquiterpene alcohol and previous study has shown that carotol may be involved in allelopathic interactions expressing activity as an antifungal, herbicidal and insecticidal agent (Fard *et al*. 2020).

It can be concluded that *G. brevis* is a good medicinal plant that may be considered as a potential for malaria drug development considering the phytocompounds present in it as well as their impressive antiplasmodial activity. It is therefore recommended that further purification and identification of pure (single) compound can be researched upon for furtherance increase in the alternative options of malaria chemotherapy.

## Figures and Tables

**Table 1:** Column chromatography of N-butanol subfractions of *Glyphaea brevis* leaves extract.

Subfractions	Combined fractions	Weight (g)	Yield (%)	Spot	RF value
SF1	1–14	0	0	-	0.00
SF2	15–24	0.21	2.1	5	0.63
SF3	25–40	0.49	4.9	5.2	0.65
SF4	41 46	0.35	3.5	6.5	0.81
SF5	47–51	0.98	9.8	5.5	0.69
SF6	52 60	0.88	11.6	6.5	0.81
SF7	61 65	0.86	8.6	5.5	0.69
SF8	66 73	0.73	7.3	3.5	0.44
SF9	74–89	0.54	5.4	3.5	0.44
SF10	90–117	0.44	4.4	3.0	0.38
SF11	118–137	0.39	3.9	4.0	0.50

*Notes:* Solvent movement = 8 cm; SF = subfraction

**Table 2:** Inhibitory concentration (IC_50_) test for chloroquine (PPC 4.47 μMol) on the parasitised patients’ blood samples.

Blood sample	IC_50_ (μMol)	Susceptibility
1	2.70	Sensitive
2	**6.56**	**Resistant**
3	1.25	Sensitive
4	1.03	Sensitive
5	**5.11**	**Resistant**
6	2.49	Sensitive
7	2.91	Sensitive
8	**6.95**	**Resistant**

*Note:* PPC = peak plasma concentration

**Table 3:** Inhibitory concentration (IC_50_) test for 3 chloroquine-resistant clinical *Plasmodium falciparum* isolates and *P. berghei*.

Samples	Antimalarial drugs/agents			N-butanol fraction	SF2	SF3	SF4	SF5	SF6	SF7	SF8	SF9	SF10	SF11

	Artemether (PPC 1.81 μmol)	Artemether/lumefantrine (PPC 53.5 μmol)	Artemisinin/piperaquine (PPC 1.40 μmol)											
**1**	0.81	24.35	1.21	7.63	4.17	4.14	2.89	1.03	3.58	3.4	2.45	4.76	3.34	5.48
**2**	0.3	**57.12** *	**1.42** *	4.08	5.81	4.78	5.54	4.02	4.4	4.1	3.88	4.58	4.5	6.69
**3**	1.54	26.92	0.90	4.04	6.51	4.77	3.58	3.69	1.96	3.68	4.64	3.6	7.81	5.09
**4**	1.7	**59.20** *	1.33	7.89	7.2	4.98	6.46	5.85	4.48	5.69	**4.32**	6.13	5.08	5.3

*Notes:* SF = subfraction; PPC = peak plasma concentration;

* Resistant (at peak plasma level of the drugs and agents. Sample 1 to 3 are *P. falciparum* isolates while sample 4 is *P. berghei* isolate.

**Table 4:** FTIR peak values and functional groups of SF8 of *G. brevis* leaves extract.

Peak	Wave value/number	Functional group	Compound class	Vibration mode	Intensity	Reference
1	838.7	C-H	Isoprenoids	Bending	Medium	(Thummajitsakul *et al*. 2020; Topalăa *et al*. 2017)
2	1017.6	-C-O-	Esters	Stretch	Medium	(Dwivedi *et al*. 2021)
3	1174.1	C-O	Acid or Ester	Stretching	Strong	(Thummajitsakul *et al*. 2020)
4, 5	1244.9, 1379.1	O-H	Phenol	Bending	Medium	(Kalaichelvi & Dhivya 2017)
6	1513.3	N–O-	Nitro compound	stretching	Medium	(Dwivedi *et al*. 2021; Easmin *et al*. 2017)
7	1602.8	C=C	Conjugated alkene	Stretching	medium	(Kalaichelvi & Dhivya 2017)
8	2832.8	C=O	Carboxylic acids	Stretching	Strong	(Gaddam *et al*. 2014)
9	2933.4	C-H	Alkanes (CH_2_)	Bend out-of-plane	Medium	(Baltacıoğlu *et al*. 2021; S.-N. GWang *et al*. 2016)
10	3306.1	O-H	Alcohol	Stretching	Strong	(Baltacıoğlu *et al*. 2021)

**Table 5:** Reported activity of compounds identified in sub-fraction 8 of N-butanol fraction of *G. brevis* leaves extract.

S/N	Name of compound	Formula	S.I. (%)	Reported biological activity	Reference
1	(-)-carvone	C_10_H_14_O	94	Antimicrobial, antitumour, antiparasitic, antineuraminidase, antioxidant, anti-inflammatory	(Bouyahya *et al*. 2021; Verstegen-Haaksma *et al*. 1995)
2	Dodecanoic acid, methyl ester	C_13_H_26_O_2_	98	Antimicrobial	(Marahatta *et al*. 2019; Taha & Mudawi 2018)
3	Carotol	O	81	Anticancer (Cytotoxic against cancer cells)	(Önder *et al*. 2021)
4	Hexadecanoic acid, methyl ester	C_17_H_34_O_2_	98	Antioxidant, anti-inflammatory, antihyperlipidemic, antibacterial, antimicrobial functions	(Enenebeaku *et al*. 2021; Nock & Amlabu 2020; Shaaban *et al*. 2021; Ukwubile *et al*. 2019)
5	9-octadecenoic acid (Z)-, methylester (oleic acid)	C_19_H_36_O_2_	99	Antimalarial, antibacterial, antioxidant, anticancer	(Nock & Amlabu 2020; Orabueze *et al*. 2020; Ukwubile *et al*. 2019; Zahara *et al*. 2022)
6	Methyl stearate	C_19_H_38_O_2_	98	Anti-inflammatory	(Adnan *et al*. 2019; Enenebeaku *et al*. 2021; Marahatta *et al*. 2019; Ukwubile *et al*. 2019)
7	Heptadecanoic acid, 16-methyl-, methyl ester	C_19_H_38_O_2_	95	Protein, anticancer	(Enenebeaku *et al*. 2021)
8	Clindamycin	C_18_H_33_ClN_2_O_5_S	83	Antioxidant, anti-inflammatory, anti-apoptotic roles	(Ibrahim *et al*. 2020)
9	Ropivacaine	C_17_H_26_N_2_O	72	Anti-(colon)-cancer	(X. Wang & Li 2021)
